# Supplementary material for: Complexome profiling of the Chlamydomonas psb28 mutant reveals TEF5 as an early PSII assembly factor
Source: Plant Cell. 2025 Mar 17;37(6):koaf055. doi: 10.1093/plcell/koaf055 (PMC12164590; doi:10.1093/plcell/koaf055)
Supplement: koaf055_Supplementary_Data [file koaf055_supplementary_data.zip › psb28_interactive.zip!/plots/intensity/qualityPlots/peptides/PFL1_Cre01.g044800.t1.2.html]

### Cre01.g044800.t1.2

Trivial name: PFL1  
SeqCoverage [median]: 35.70  
Mass: 91.10 kDa  
  
 

| Sequence | MissedCleavage | Mass [Da] | wtIntensity | muIntensity | Score |
| --- | --- | --- | --- | --- | --- |
| AALESYGYTPDPEVAR | 0 | 1737.82 | 4039.27 | 22513.25 | 187.85 |
| AIKPLGGVNMVK | 1 | 1225.72 | 18827.36 | 40259.41 | 119.27 |
| ALSELKEMGAAYGFDLSRPAANSR | 2 | 2553.26 | 0.00 | 1209.35 | 56.05 |
| DAHGALASLNSVAK | 0 | 1352.70 | 1480.11 | 16798.63 | 154.74 |
| EQQLEVIAR | 0 | 1084.59 | 2319.17 | 5962.52 | 84.50 |
| GLMTDFKVEGSFPK | 1 | 1554.78 | 0.00 | 14781.09 | 116.84 |
| HNLLGVMDEEKIR | 1 | 1552.80 | 0.00 | 456.44 | 64.12 |
| IDAFLDTYFER | 0 | 1388.66 | 348.05 | 0.00 | 55.40 |
| KGEPFAPGANPLHGR | 1 | 1546.80 | 0.00 | 1141.33 | 35.66 |
| KKGEPFAPGANPLHGR | 2 | 1674.90 | 0.00 | 3782.67 | 59.86 |
| KSGILSGLPDGYGR | 1 | 1418.75 | 0.00 | 2157.49 | 31.83 |
| LLLYTLNGGRDEVSGDQVGPK | 1 | 2230.16 | 0.00 | 6349.80 | 94.28 |
| LREEVNEQIR | 1 | 1284.68 | 17516.14 | 52049.85 | 145.46 |
| LTREQQLEVIAR | 1 | 1454.82 | 0.00 | 3573.80 | 113.24 |
| LYSTVR | 0 | 737.41 | 0.00 | 1437.00 | 85.31 |
| PKPAVDAGINVQK | 1 | 1335.75 | 2142.71 | 1476.73 | 69.35 |
| SGILSGLPDGYGR | 0 | 1290.66 | 403.88 | 7867.34 | 102.52 |
| SMLMDAVEHPEKYPNLTIR | 1 | 2243.11 | 0.00 | 7596.93 | 62.20 |
| SPTAPLDYEEVK | 0 | 1347.66 | 312.38 | 2948.22 | 123.79 |
| SPTAPLDYEEVKAK | 1 | 1546.79 | 5740.88 | 9557.33 | 92.94 |
| TFHDTM | 0 | 750.30 | 538.47 | 478.90 | 113.82 |
| VALYGVDALIK | 0 | 1160.68 | 7193.87 | 8754.63 | 99.80 |
| VGKDMQYFGAR | 1 | 1270.61 | 977.11 | 1783.07 | 51.09 |
| VSGYAVHFAR | 0 | 1105.57 | 0.00 | 8547.36 | 92.05 |
| VSLDTSSIQYESDNLMSK | 0 | 2015.94 | 1039.09 | 8903.98 | 102.52 |
| YAQVTPVIDER | 0 | 1289.66 | 5731.80 | 13236.95 | 160.53 |
|  |  |  | 68610.3 | 243624.1 |  |
